# A Family with a High Incidence of Migraine and Vestibular Migraine and a Case of Menière's Disease

**DOI:** 10.1155/2021/9984047

**Published:** 2021-09-02

**Authors:** Marco Familiari, Omar Gatti, Iacopo Cangiano, Roberto Teggi

**Affiliations:** Department of Otolaryngology Head & Neck Surgery, IRCCS San Raffaele Scientific Institute, Milan, Italy

## Abstract

Vestibular migraine (VM) and Menière's disease (MD) are common neurotological disorders causing episodic vertigo. Sometimes, VM is accompanied by cochlear symptoms suggestive for MD. Therefore, in those cases, the differential diagnosis between the two disorders can be difficult. Moreover, a comorbidity with migraine in MD patients is widely reported, up to the hypothesis of a possible MD-VM overlapping syndrome. In this brief case report, we consider the clinical history of a family presenting high incidence of subjects fulfilling the diagnostic criteria of VM and single case fulfilling criteria for definite MD. The relationship between VM and MD is still under debate; anyway, it can be speculated that commonly shared genetic mutations could play a role as predisposing factors in both disorders. A congenital nystagmus in the family was present too, but its correlation with the other conditions is still not clear. Future goal of our work will be to assess genetics in this family.

## 1. Introduction

Vestibular migraine (VM) is a very common neurotological disorder affecting 1-2% of the European population, whose diagnostic criteria, based on clinical history, have recently been drawn up by a joint committee of the Barany Society and International Headache Society [1].

The followings are criteria for definite VM:

(A) At least five episodes with vestibular symptoms of a moderate or severe intensity, lasting from 5 minutes to 72 hours
(B) Current or previous history of migraine with or without aura according to the International Classification of Headache Disorders (ICHD)
(C) One or more migraine features with at least 50% of the vestibular episodes
  - Headache with at least two of the following characteristics: one side location, pulsating quality, moderate or severe pain intensity, and aggravation by routine physical activity;
  - Photophobia and phonophobia
  - Visual aura
(D) Not better accounted for by another vestibular or ICHD diagnosis

In order to diagnose probable VM, only one of the criteria B or C must be observed. For probable VM, only one of B/C criteria should be fulfilled.

Recently, some authors have tried to highlight its clinical characteristics, noting, however, a strong heterogenicity of symptoms and phenotypes; above all, according to some authors, cochlear symptoms such as fluctuating hearing loss, tinnitus, or fullness have been reported by several patients during VM attacks [2, 3]. As a consequence, in early stages, a differential diagnosis between VM and Menière's disease (MD) may be difficult [4].

On the other side, among MD subjects, a comorbidity with migraine is far from being rare, ranging from 40 to 50% of patients [5, 6].

The overlap of vertigo attacks in the same patient with features of VM and MD for duration of attacks and accompanying symptoms has been reported by some authors, who proposed for the condition the name of VM/MD overlapping syndrome [7].

Genetic factors have been studied as a predisposing factor for both MD and VM, although at present, results are inconclusive, and normally, signs and symptoms of the diseases tend to appear at an earlier age as the disorder is passed from one generation to the next (anticipation) [8]; curiously, in one work, it has been reported that 66% of VM patients referred another relative in the first or second degree suffering from vertigo, and when asked about the known diagnosis 7.1% referred MD [2], underlying the possibility of commonly shared genetic predisposing factors between the two disorders [9].

The aim of this work is to describe the case of a family presenting a high incidence of subjects presenting episodic vertigo fulfilling the diagnostic criteria for definite VM and a single case presenting episodic vertigo fulfilling criteria for definite MD.

## 2. Clinical History

**Figure 1:**
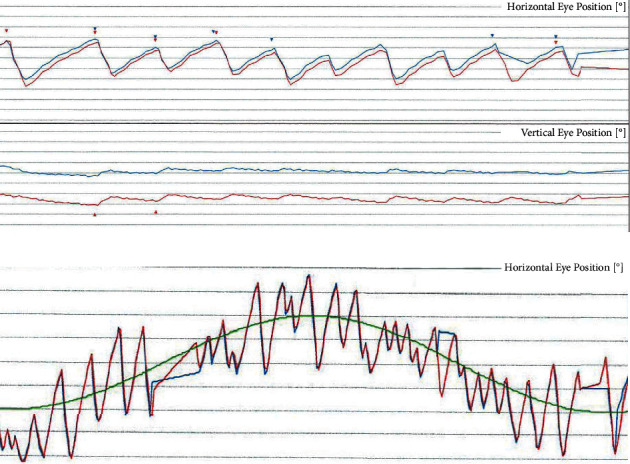
The records of spontaneous nystagmus of the first sister in the primary position (a) and during execution of pursuits (b).

The first sister, aged 14, presented a smooth horizontal conjugate left beating nystagmus, according to the parents congenital; the frequency of nystagmus increased during execution of pursuits, during which the two phases seemed to be of equal velocity (Figure 1), and decreased during movements of convergence, but the patient did not refer oscillopsia.

She was studied previously by neurologists and ophthalmologists without conclusive results; she does not suffer from albinism. When six-year-old, she suffered from torticollis, while till present, she had motion sickness. At the age of 12, she began to suffer from internal vertigo attacks lasting around 30 minutes and normally followed by headaches with migrainous features (lasting till 1 day, with photo and phonophobia, worsening with efforts and located around one eye and spreading to the left hemicranium, without aura). Vertigo was accompanied by nausea/vomiting, but no cochlear symptoms were reported. Rather than for the presence of congenital nystagmus, she fulfilled criteria for definite vestibular migraine. The frequency of vertigo spells was around one month, more frequently in the catamenial period. As a consequence of the congenital nystagmus, pursuits presented an almost complete saccadization, and vestibular assessment was poorly reliable. She had a normal audiometric threshold.

**Figure 2:**
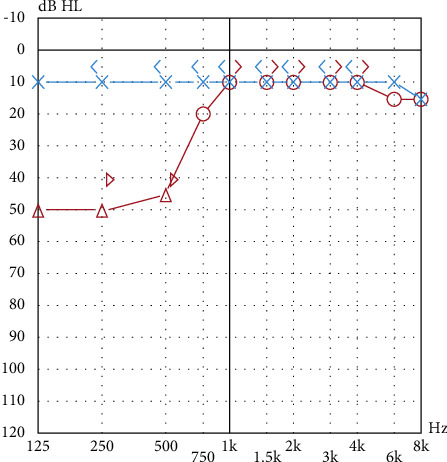
The audiogram showing low to medium frequencies sensorineural hearing loss on the second sister's right ear.

The second sister, aged 23, suffered of abdominal pain and motion sickness when she was around 4. From the age of 12, she suffered from headache with migrainous features (pulsatile and accompanied by photo and phonophobia, without aura) without vertigo, lasting till two days. At the age of 18, she began to report vertigo attacks lasting several hours with vomiting, fullness, and fluctuating hearing loss on the right side, not associated with migrainous symptoms. She reported a frequency of one attack every 6 months, more frequently, but not always in the catamenial period. An audiometric exam demonstrated low to medium frequencies sensorineural hearing loss on the right side with a difference in threshold of 40 decibel (dB) between 250 and 1000 Hertz (Hz) suggestive for a hydropic disorder (Figure 2). A vestibular bedside examination (including video head impulse, head shaking, skull vibration, and positional tests and eye movements evaluation) was negative rather than for the presence of a left beating nystagmus during the skull vibration test.

Both sisters performed a central nervous system MRI, always negative for any pathological condition.

The father, aged 57, presented a history of migrainous headache from the age of 30, without aura and sporadical attacks of dizziness from the age of 35, without a sure correlation with headache. He suffered from hypertensive disorders anyhow well controlled with beta-blockers. An audiometric exam showed a bilateral and symmetric high frequency hearing loss, compatible with presbycusis. He fulfilled criteria for probable VM.

The mother, aged 47, suffered from benign paroxysmal vertigo in pediatric age (around 4-5 years old); she had migrainous headaches (without aura) from the age of 23 and in the last three years also sporadic vertigo lasting not more than 30 minutes often accompanied by a long lasting (till one day) headache and nausea. The bedside examination demonstrated normal pursuits and saccades and a positive skull vibration test. A central nervous system magnetic resonance imaging (MRI) showed microischemic lesions.

The mother had one sister and two brothers. The sister, aged 50, reported a positive history for migrainous headache, whose onset was at around 25; she also reported short episodes of dizziness/internal vertigo lasting 10 minutes since she was 35-year-old, more frequently during headaches, while some episodes of migrainous headache were preceded by eye flashes of light lasting minutes (compatible with aura). Central nervous system MRI highlighted microischemic lesions.

One of the two brothers, aged 52, also suffered from migrainous headache (without aura), whose onset was at 30. The other brother, aged 53, had no pathological disorder except for a mild hypertension well controlled with diuretic.

**Figure 3:**
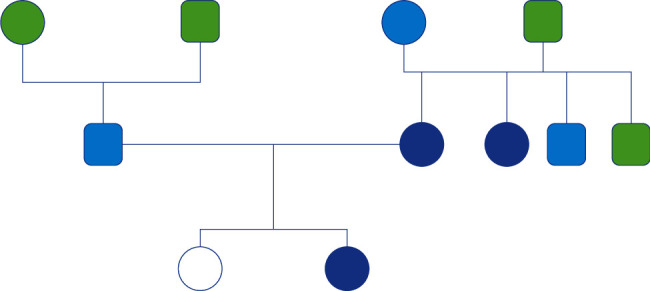
Pedigree of the family starting from the grandparents. The circles represent women; the squares represent men; the green color represents healthy people who does not fulfill criteria for vestibular migraine or Ménière's disease; the light blue color represents people with the history of migraine without vertigo; the blue color represents people who fulfills criteria for vestibular migraine; the white color represents people who fulfills criteria for definite Ménière's disease.

No affordable data were found on grandparents, except that maternal grandmother also suffered from headaches from the age of 25. A pedigree describing the distribution of the pathologies in this family is shown in Figure 3.

## 3. Discussion

Some speculations can be made concerning this family case. Our family presents a high rate of VM, since one sister and parents presented a clinical history of VM (definite or probable). The aunt also satisfied diagnostic criteria for definite VM.

Moreover, considering the onset of both migraine and vestibular migraine, an anticipation of the onset of both migrainous headache and vertigo should be underlined [10]; second, both daughters reported an history of migraine precursors (torticollis and motion sickness in the younger; abdominal pain and motion sickness in the other); similarly, the mother reported short episodes of dizziness resembling benign paroxysmal vertigo of childhood [2].

The particular feature of this case is the onset of a MD phenotype in a migraineur woman, whose family presented phenotypes of migraine and definite (the sister and mother) or probable (the father) VM.

As a first consideration, it is still under debate if MD is a single disorder or a burden of symptoms, and recently, some authors proposed to differentiate five groups of patients with unilateral and five with bilateral MD according to a cluster analysis of comorbidities [11, 12]. Among these groups, the subgroups of subjects with migraine presented a lower age of onset of vertigo.

The relationship between the two disorders is at present under debate. For example, it has been reported that VM attacks may be accompanied by cochlear symptoms [3]. On the other hand, some authors proposed the possibility of a simultaneous presentation of the disorders [7], and the case of a contemporary presentation of migraine, MD, and other episodic vertigo in six families has been published some years ago [13].

Since for both VM and MD a genetic predisposition has been proposed [8], it can be speculated that a commonly shared genetic variance may be the reason of overlapping of vertigo phenotypes. Among the possibilities, ionic transporters have been proposed as a predisposing factor for both migraine and MD, and it can be speculated that this is the reason of the overlapping in the family of VM and MD phenotypes [9]. In particular, Oh et al. identified a mutation involving *TRPM7* gene encoding Ca2+ and Mg2+ ionic channels in a family suffering from VM [14].

However, this is not the first report about this topic. In fact, Requena et al. reported a case of probable VM in a family with familial MD [15], whereas Martìn-Sierra et al. reported several cases of migraine not cosegregated with MD in a family suffering from the same disorder [16]. Moreover, in a recent study, VM patients showed to have a positive familial history for MD in 7.1% of cases [2].

The association between infantile-congenital nystagmus in one of the daughters, migraine, and MD is not clear, but their concomitant presence in the same family is very interesting. As far as we know, excluding neurological disorders, a single X-linked gene, *FRMD-7*, has been proposed as a candidate for the disorder, encoding a protein, the FERM domain-containing protein 7, which plays an important role in neuronal development and is involved in the regulation of F-actin, although its specific mechanism of action remains undetermined [17].

In conclusion, a single subject in the migraineur family presents a different phenotype coherent with MD, suggesting the possibility that common genetic mutations could play a relevant role as predisposing factors in both disorders. A case of congenital nystagmus was present too, but its correlation with other conditions is still not clear. Future goal of our work will be to assess genetics in this family.
